# Whole-Genome Sequencing of Pathogenic *Nigrospora musae* ST1 Causing Leaf Spot Disease in *Idesia polycarpa*

**DOI:** 10.3390/jof12030226

**Published:** 2026-03-19

**Authors:** Yun-Ze Chen, Yan Chen, Jing Yang

**Affiliations:** 1 College of Biological Sciences, Guizhou Education University, Gaoxin Road 115, Guiyang 550018, China; 2 College of Forestry, Guizhou University, Guiyang 550025, China; chenyan66882025@outlook.com (Y.C.); jingyang@gzu.edu.cn (J.Y.)

**Keywords:** *Idesia polycarpa*, *Nigrospora musae* ST1, fungal pathogens, whole-genome sequencing, genome annotation

## Abstract

*Nigrospora musae* ST1 is a newly identified pathogen responsible for leaf spot disease in *Idesia polycarpa*. In order to further advance our understanding of this strain and improve management strategies for the leaf spot disease, the PacBio Sequel II platform was used to perform whole-genome sequencing of *N. musae* ST1. The assembled genome comprised 42 contigs, with a total length of 49,259,803 bp and an average GC content of 56.23%. Functional annotation identified 12,063 protein-coding genes, including 125 Transporter Classification Database (TCDB)-related genes, 3600 pathogen host interaction (PHI) genes, 2503 Virulence Factor Database (DFVF)-related genes, and 722 genes encoding carbohydrate-active enzymes (CAZymes). Integrated analyses of the secretome, PHI, and DFVF databases revealed six secreted carbohydrate-active enzymes implicated in plant pathogenicity, including three glycoside hydrolases, two pectinate lyases, and one cutinase, potentially playing important roles in pathogenicity. A total of 77 secondary metabolite gene clusters were predicted. Comparative genomic analysis between *N. musae* ST1 and other *Nigrospora* species revealed differences in genome rearrangements in *Nigrospora* fungi. In conclusion, this study has clarified the whole-genome structural characteristics and evolutionary relationships of the newly reported pathogenic fungus, *N. musae* ST1. It provides a theoretical foundation for future investigations into the pathogenic mechanisms of *N. musae* ST1 infection in *I. polycarpa*, as well as potential targets for disease control.

## 1. Introduction

*Idesia polycarpa* is an important oil-producing woody plant, extensively distributed across the southwest, eastern, and southern China. Due to its high seed oil content and large proportion of unsaturated fatty acids, it has a wide application potential for biodiesel production and the food industry, representing an energy crop and economically valuable tree species with significant development prospects [1,2]. In recent years, *I. polycarpa* has been designated as a key economically important tree species for the development of specialty industries in Guizhou Province, which is of great significance in promoting the economic development of mountainous regions. However, with the expansion of large-scale cultivation of *I. polycarpa*, pathogenic fungi could seriously affect its normal growth, thereby reducing its fruit yield. In preliminary investigations, a previously unreported pathogen, *N. musae*, was isolated and identified as the causal agent of *I. polycarpa* leaf spot disease [3], posing a new challenge to its cultivation.

*N. musae* belongs to the kingdom Fungi, phylum Ascomycota, class Sordariomycetes, order Amphisphaeriales, family Apiosporaceae, and genus *Nigrospora*. Species within this genus comprise beneficial plant endophytes as well as pathogenic species capable of infecting both humans and plants [4]. In particular, in recent years, plant leaf blight caused by fungi of the genus *Nigrospora* has become increasingly common, affecting a wide range of hosts such as *Sesame* [5], *Ginger* [6], *Poplar* [7], *Platycodon grandiflorus* [8], *Basella alba L* [9], *I. polycarpa* [3], and *broad bean* [10]. Additional diseases attributed to *Nigrospora* include southern yew stem blight [11], *Akebia trifoliata* Fruit Dried-Shrink Disease [12], *Siberian Lily* (*Lilium* spp.) Basal Rot [13], *Mulberry Shot* Hole, and others [14]. It severely affects plant growth and crop yield, and poses a great threat to agricultural production.

Advances in sequencing technologies have enabled whole-genome sequencing to provide comprehensive genomic information on pathogens, including the number of genes, genes encoding secondary metabolites, plant cell wall-degrading enzymes, and so on [15]. Studies have shown that whole-genome sequencing and assembly of pathogenic fungi can help identify the key pathogenic determinants in fungal pathogens. For example, studies on *Ustilago crameri* [16] and *Talaromyces albobiverticillius* [17] have shown that secondary metabolites of pathogenic fungi are closely associated with the induction of plant cell death. At present, genomic data for 16 *Nigrospora* species are available in the NCBI database. However, the genome resources of *Nigrospora* plant pathogenic fungi are limited, and research on pathogenic genes and their pathogenic mechanisms is still scarce. Whole-genome sequencing thus provides an essential foundation for elucidating the molecular basis of pathogenicity in plant pathogens.

In view of this, we report the genome of the fungus that causes *I. polycarpa* leaf spot, providing genomic resources for studying the pathogenesis of *Nigrospora*.

## 2. Materials and Methods

### 2.1. Fungal Material and Culture Conditions

The pathogenic fungus was isolated from *I. polycarpa* leaves exhibiting leaf spot blight symptoms collected from GuiDing (PQ549970.1, PQ559744.1, PQ559741.1), GuiZhou Province, China, and preserved at the College of Biological Sciences, Gui Zhou Education University (Guiyang, China). The strain was cultured in potato dextrose broth at 25 °C for 2 days.

### 2.2. DNA Extraction

The mycelium of *N. musae* ST1 was obtained from cultures grown in 200 mL of fresh potato dextrose broth (PDB) at 28 °C for 2 days. Hyphae were aseptically filtered and washed thoroughly with sterile water. Genomic DNA was then extracted using QIAGEN Genomic-tip 10,262 (Qiagen, Inc., Norcross, GA, USA).

### 2.3. Whole-Genome Sequencing

Whole-genome sequencing of *N. musae* ST1 was performed by Biomarker Technologies Corporation (Beijing, China). Raw reads were subjected to quality filtering using SMRT Link v5.0.1 (https://www.pacb.com/smrt-link/, accessed on 20 June 2025) to remove low-quality sequences (reads < 500 bp), yielding high-quality clean reads. Genome assembly was conducted de novo using the automatic error correction function of the SMRT portal website [18,19]. Hifiasm v0.12-r304 [20] software was used to assemble the filtered reads, and Pilon v1.22 [21] software was used to further correct the assembly genome to obtain a genome with higher accuracy. Finally, the integrity of the genome assembly was evaluated by using BWA v0.7.17 software [22] and BUSCO v2.0 software [23].

### 2.4. Transcriptome Sequencing

The experimental samples were sent to Biomarker Technologies Co., Ltd. (Beijing, China) for RNA extraction and cDNA library construction. After passing quality control, libraries were sequenced on the Illumina platform to ensure data quality and accuracy. Clean Reads were accurately aligned to the reference genome using HISAT2 [24] to obtain mapping information of the reads on the genome. The mapped reads were assembled using StringTie v1.2.3 [25]. Gene structure refinement, as well as novel gene prediction, were conducted by comparing the assembled transcripts with the reference genome. The expression levels of genes were calculated using the StringTie [26] tool, with FPKM used as the expression metric.

### 2.5. Genome Component Prediction

Based on the structural prediction and de novo prediction methods, the genome repeat sequence database was constructed using LTR\U FINDER v1.05 [27], MITE Hunter [28], RepeatScout v1.0.5 [29], and PILER-DF v2.4 [30]. The database was classified with PASTEClassifier [31], and then combined with the Repbase [32] database to generate the final database. RepeatMasker v4.0.6 [33] was used for repeat annotation. Gene structure prediction incorporated de novo prediction, homologous protein prediction, and transcriptome evidence prediction, and then the three prediction results were integrated. Genscan [34], Augustus v2.4 [35], GlimmerHMM v3.0.4 [36], GeneID v1.4 [37], and SNAP (version 2006-07-28) [38] were used for de novo prediction. Homologous protein prediction was performed using GeMoMa v1.3.1 [39]. Hisat2 v2.0.4 [40] and Stringtie v1.2.3 [40] were used for reference transcript-based assembly. TransDecoder v2.0 [41] (Haas) and PASA v2.0.2 [42] were used to predict Unigene sequences based on transcriptome assembly. Finally, EVM v1.1.1 [41] was used to integrate the prediction results obtained by the above three methods, followed by refinement with PASA v2.0.2. tRNA prediction was performed using tRNAscan-SE v1.3.1 [43], and Infernal 1.1 [44] was used to predict rRNAs and other ncRNAs other than tRNAs and rRNAs, based on the Rfam v12.0 [45] database. Circos [46] was used to generate circular genome visualizations. GenBlastA [47] was used to search for homologous gene sequences, and pseudogenes were detected using GeneWise v2.2.0 [48] to identify premature stop codons and frameshift mutations. Secondary metabolite biosynthetic gene clusters were predicted using antiSMASHv6.0.0 [49].

### 2.6. Gene Function Annotation

The predicted proteins were annotated using blast [50] (e-value: 1 × 10^−5^) against Clusters of orthologous groups (KOG, https://ftp.ncbi.nlm.nih.gov/pub/COG/KOG/, accessed on 21 June 2025) [51], Kyoto Encyclopedia of Genes and Genomes (KEGG, https://www.genome.jp/kegg/, accessed on 21 June 2025) [52], Swiss Protein Sequence Database (Swiss-Prot protein knowledgebase, http://www.expasy.org/sprot/, accessed on 21 June 2025) [53], Translation of EMBL (TrEMBL, http://www.ebi.ac.uk/embl/index.html, accessed on 21 June 2025) [54], and Non-Redundant Protein Sequence (Nr) databases (https://ftp.ncbi.nlm.nih.gov/blast/db/FASTA/nr.gz, accessed on 21 June 2025) [55]. Blast2go [56] was used for Gene Ontology (GO) annotation. Hmmer [57] was used for Pfam [58] annotation. Further gene function annotation analysis included COG and KEGG metabolic pathway enrichment analysis and GO functional enrichment analysis. Pathogenicity-related genes were identified by BLAST [54] searches against the TCDB (https://www.tcdb.org/, accessed on 21 June 2025) [59], PHI (www.phi-base.org, accessed on 21 June 2025) [60], CYPED (http://www.cyped.uni-stuttgart.de, accessed on 21 June 2025) [61], and DFVF (http://sysbio.unl.edu/DFVF/, accessed on 21 June 2025) [62] databases. In addition, hmmer [57] was used to perform functional annotation of carbohydrase genes based on the CAZy database (https://www.cazy.org/, accessed on 21 June 2025) [63]. Protein sequences of all predicted genes were analyzed using SignalP 4.0 [64] to identify proteins containing signal peptides. All predicted protein sequences were analyzed using TMHMM 2.0c [65] to identify proteins containing transmembrane helices. Proteins containing both signal peptides and transmembrane helices were excluded to obtain the final secretome. Secreted proteins were further analyzed by EffectorP [66] to predict fungal effector proteins. Proteins longer than 300 amino acids were removed prior to PHI database comparison (http://phi-blast.phi-base.org, accessed 21 June 2025). Functional domain composition was analyzed using NCBI CDD (https://www.ncbi.nlm.nih.gov/cdd, accessed 21 June 2025), and the results were visualized with TBtools v2.056.

### 2.7. Genome-Level Comparison with Other Nigrospora Species

The available chromosome-level and complete genome assemblies of *Nigrospora* species (*Nigrospora musae* Guizhou (accession number: JAQZDL000000000), *Nigrospora oryzae* GZL1 (accession number: JADKYO000000000), *Nigrospora sphaerica* ZJJ_C1 (JAGVVL000000000), *Nigrospora guilinensis* LC3481 (accession number: JAQZDO000000000), *Nigrospora hainanensis* LC7030 (accession number: JAQZDN000000000), *Nigrospora lacticolonia* LC3324 (accession number: JAQZDM000000000), *Nigrospora osmanthi* Z1 (accession number: GCA_029489945.1), *Nigrospora pyriformis* LC2045 (accession number: JAQZDJ000000000), *Nigrospora bambusae* LC7114 (accession number: JAQZDR000000000)) were used for comparative analysis. fastANI 1.33 was used for ANI analysis. The online website D-Genies (https://dgenies.toulouse.inra.fr/, accessed 10 November 2025) was used for genome-level collinearity analysis.

### 2.8. Data Availability

The *N. musae* ST1 genome sequence was deposited in NCBI under accession number JBSLLA000000000. The genome raw sequencing data and the assembly reported in this paper are available in the NCBI BioProject: PRJNA1367030, and BioSample: SAMN53328914 within GenBank. The SRA accession number is SRR36218717.

## 3. Results

### 3.1. Genomic Characteristics

The circular consensus sequencing (CCS) reads of *N. musae* ST1 were sequenced using the PacBio Sequel II sequencing platform and assembled de novo. The CCS read data were 2.73 G, the complete genome size was 49.25 MB, and the sequencing depth was 55.44×. Hifiasm software was used to assemble CCS reads, and Pilon software was used to further correct the assembled genome using second-generation sequencing data.

**Figure 1:**
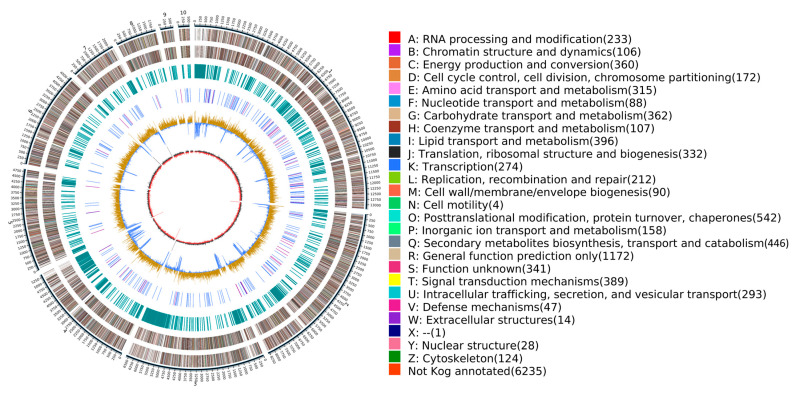
Genome circle diagram of *N.musae* ST1. The outermost circle represents the location coordinates of the contig 1–10 of the genome sequence.

The total length of the final assembled genome was 49,259,803 bp with a GC content of 56.23%. The assembly consisted of 42 contig numbers with no gaps. The N50 length was 6,617,117 bp, and the N90 length was 2,194,971 bp (Table 1). The entire genome atlas of *N. musae* ST1 was generated using Circos (v 0.69-8) (Figure 1).

*N. musae* ST1 genome completeness was assessed using both next-generation read mapping and BUSCO analysis. An evaluation of next-generation sequence data recovery provided an alignment ratio of 94.56%, indicating 99.97% sequence coverage of the assembled genome and a final sequencing depth of 69.81×. BUSCO analysis revealed 287 complete BUSCO genes, indicating a genome completeness of 98.97%, confirming the high quality of the obtained genome assembly (Table 2).

### 3.2. Sequence Prediction

#### 3.2.1. Repeat Sequence, Protein-Coding Gene, Non-Coding RNA, and Pseudogene Prediction

Repetitive elements were classified into major repeat categories and subfamilies. The genome contained 2,565,439 bp of repetitive sequences, accounting for 5.21% of the total genome.

**Figure 2:**
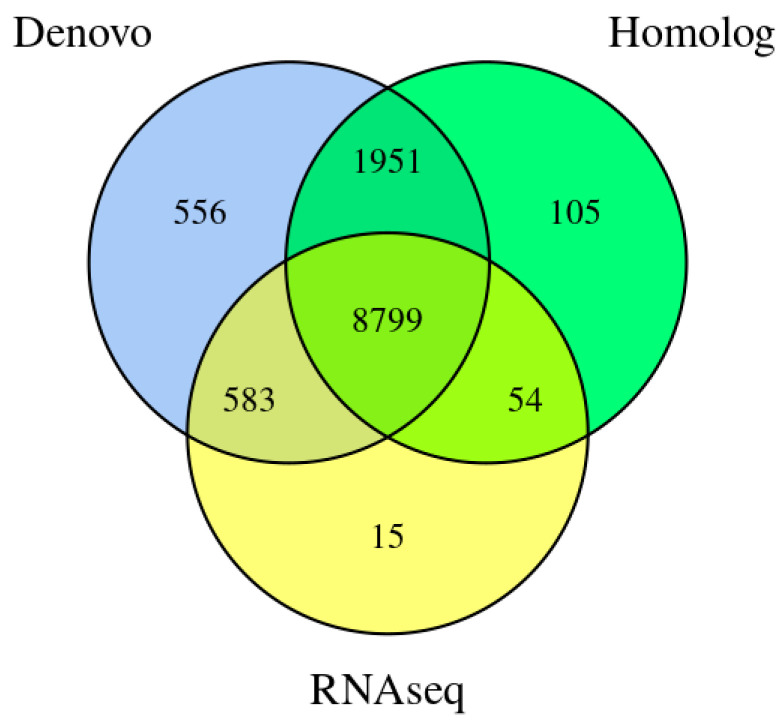
Integrated genes of *N. musae* ST1 derived from three prediction methods.

Using a combination of de novo prediction, homologous protein prediction, and transcriptomic evidence, we identified a total of 12,063 protein-coding genes in the genome of *N. musae* ST1. Among these, 11,507 genes (95.39%) were supported by homology prediction and transcript evidence, indicating high annotation reliability. The total length of the encoded genes was 25,915,843 bp, accounting for 52.61% of the whole genome. The average number of exons per gene was 2.95; the average number of CDSs per gene was 2.88; and the average number of introns per gene was 1.95 (Table A1 and Figure 2).

The results of the genomic data of *N. musae* ST1 revealed 475 non-coding RNAs, including 78 ribosomal RNAs (rRNAs), 332 transfer RNAs (tRNAs), and 75 other non-coding RNAs. The rRNAs comprised 5 5.8S_rRNAs, 6 28S_rRNAs, 18 18S_rRNAs, and 49 5S_rRNAs. A total of 17 pseudogenes containing premature stop codons or frameshift mutations were detected, with a combined length of 65,191 bp and an average length of 3834.76 bp.

#### 3.2.2. Prediction of Gene Clusters

The analysis of secondary metabolite gene clusters using the AntiSMASH tool identified a total of 77 gene clusters with a combined length of 3,225,987 bp and an average length of 41,895 bp (Table 1). These included 14 terpene, 29 T1PKS, 4 indole, 6 NRPS-like, 6 NRPS, 6 NRPS-Like, 5 NRPS + T1PKS, 4 NRPS-Like + T1PKS, 1 T3PKS, 1 fungal-RiPP, 1 T1PKS + NRPS-like, 1 T1PKS + NRPS, 1 NRPS-like + indole, 1 T1PKS + terpene, 1 NRPS + T1PKS + indole, 1 Betalactone + T1PKS + NRPS-like, and 1 NRPS + NRPS-like clusters. Only 19 BCGs showed homologies with known clusters, which are putatively responsible for the biosynthesis of compounds such as alternapyrone, dimethylcoprogen, serinocyclin, depudecin and betaenone (Table A2). Among them, three BCG showed 100% similarity to known clusters (Table A2). Approximately 96.10% of clusters showed no similarity to characterized BGCs, suggesting that *N. musae* ST1 may produce numerous previously unknown secondary metabolites.

**Figure 3:**
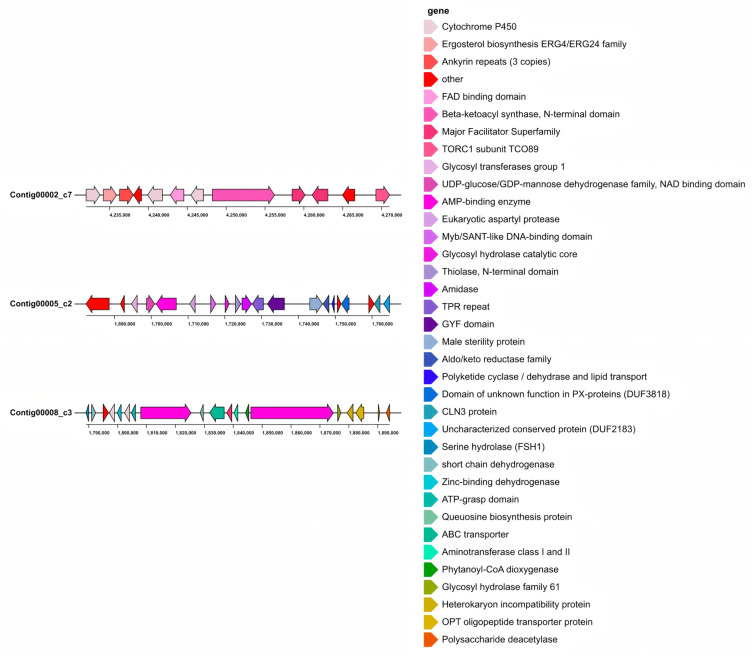
Three identified BGCs with high identity in *N. musae* ST1. Region 2.7 had a T1PKS gene cluster; region 5.2 had two NRPSs and an NRPS-like gene cluster; region 8.3 had an NRPS and TIPKS gene cluster.

Region 2.7 includes a BGC of alternapyrone and a PKS gene (GenBank: AB120221.1A). Alternapyrone was first discovered in the potato *Alternaria solani* [67]. As a secondary metabolite of fungi, alternapyrone exhibits cytotoxicity and phytotoxicity (Figure 3) [68,69].

Region 5.2 exhibits significant similarity with the BGC of *Alternaria alternata* (GenBank: JQ973666.1). Dimethylcoprogen is an extracellular siderophore complex produced by pathogenic fungi under iron-deficient conditions [70,71]. It can enhance the utilization of iron by fungi to promote fungal growth and control the growth of other microorganisms in the environment (Figure 3) [72].

Region 8.3 contains serinocyclin gene clusters (BGCs) capable of synthesizing two compounds, serinocyclin A and serinocyclin B, classified as a terpene cluster (GenBank: JELW01000052.1). Structurally, serinocyclin A (1) contains three serine units, one hydroxyproline (Hyp), beta alanine (beta Ala), and two uncommon non-proteinogenic amino acids, 1-aminocyclopropane-1-carboxylic acid (Acc) and gamma hydroxylysine (HyLys). The Lys in serinocyclin B (2) replaces the HyLys unit in 1. Notably, serinocyclin is a fungal toxin, and these compounds are toxic to insects (Figure 3) [73].

**Figure A1:**
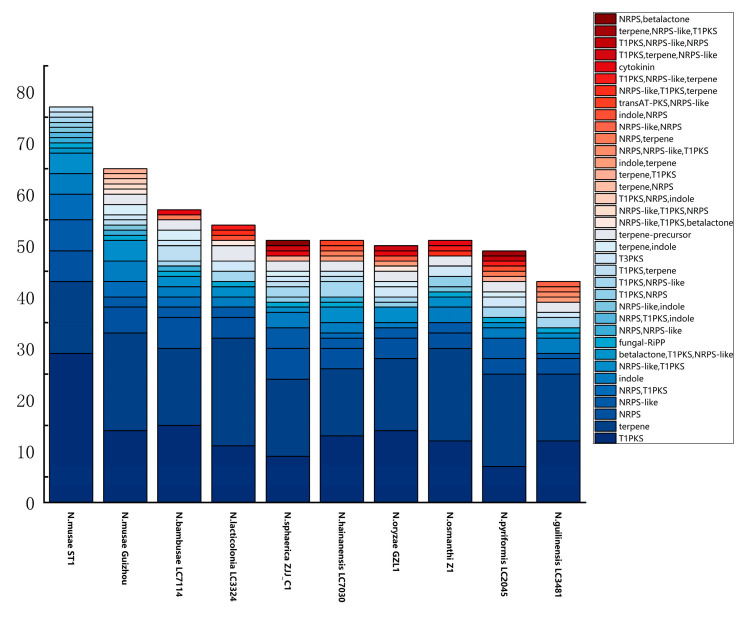
Gene cluster analysis of *Nigrospora* pathogenic fungi.

Comparative analysis of *Nigrospora* genomes revealed that *N. musae* ST1 possessed the highest number of BGCs among the species examined, followed by *N. musae* Guizhou and *N. sphaerica*. All analyzed genomes were enriched with T1PKS gene clusters and terpene gene clusters. However, substantial differences were observed in the composition and abundance of BGCs among species, indicating differences between the genomes of different plant pathogenic fungi within the genus (Figure A1).

### 3.3. Functional Annotation of the Genome

**Figure 4:**
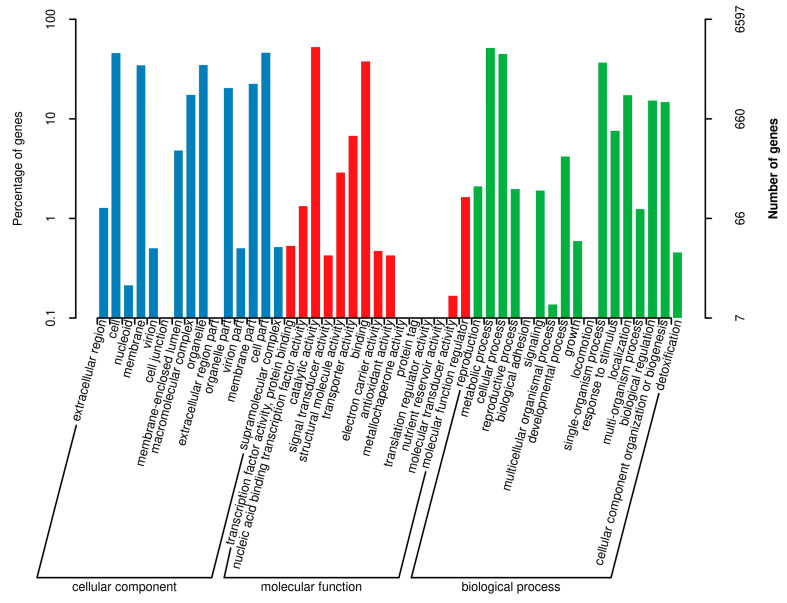
Gene Ontology (GO) functional annotation of the *N. musae* ST1 genome. The GO classification is presented with horizontal coordinates, while the percentage (**left**) and numbers (**right**) of genes are shown on the vertical axis.

Functional annotation using the GO database (Blast2go v2.5) assigned annotations to 6597 protein-coding genes in the genome of *N. musae* ST1, corresponding to 47 GO terms, and representing 54.68% of the total predicted genes (Figure 4). Within the Biological Process (BP) category, 17 GO terms were assigned, with the top three terms being “metabolic process”, “cellular process”, and “single-organism process”; within the molecular function (MF) category, 15 GO terms were annotated, with the top three terms being “catalytic activity”, “binding”, and “transporter activity”; within the Biological Process (CC) category, 15 GO terms were annotated, with the top three terms being “cell”, “organelle”, and “cell part”.

**Figure 5:**
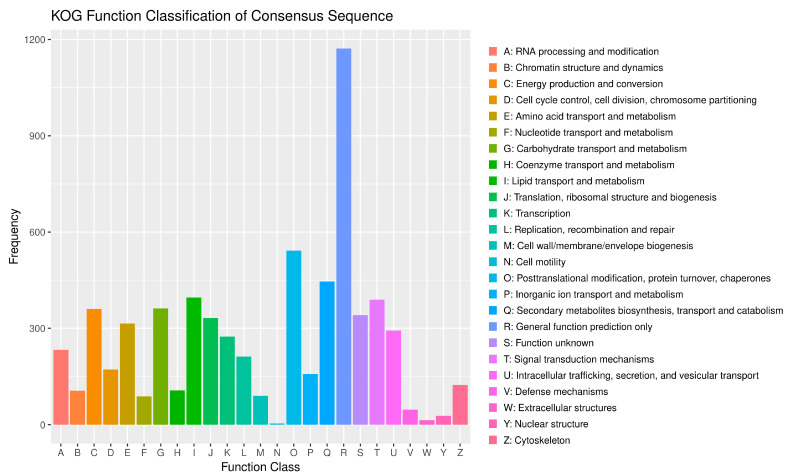
KOG annotation of *N. musae* ST1.

The number of annotated genes for *N. musae* ST1 in the KOG database was 5828, accounting for 48.31% of all predicted genes (Figure 5). The KOG category with the highest number of annotated genes was “General function prediction only”, with 1172 genes, followed by “Posttranslational modification, protein turnover, chaperones” with 542 genes, “Secondary metabolites biosynthesis, transport and catabolism” with 446 genes, “Lipid transport and metabolism” with 396 genes, “Signal transduction mechanisms” with 389 genes, “Energy production and conversion” with 362 genes, “Carbohydrate transport and metabolism” with 360 genes, and “Function unknown” with 341 genes.

**Figure 6:**
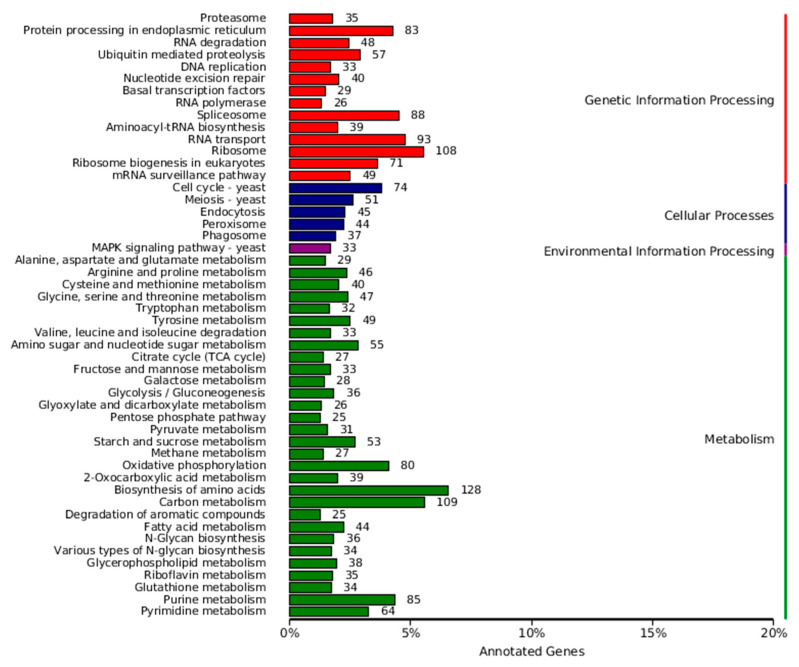
KEGG annotation classification statistics of *N. musae* ST1.

Based on the Kyoto Encyclopedia of Genes and Genomes (KEGG) pathway database, 3365 genes were annotated into 113 KEGG pathways (Figure 6). The six KEGG pathways with the highest number of assigned genes were Biosynthesis of amino acids, Carbon metabolism, Ribosome, RNA transport, Spliceosome, and Purine metabolism.

### 3.4. Carbohydrate-Active Enzymes (CAZymes)

**Figure A2:**
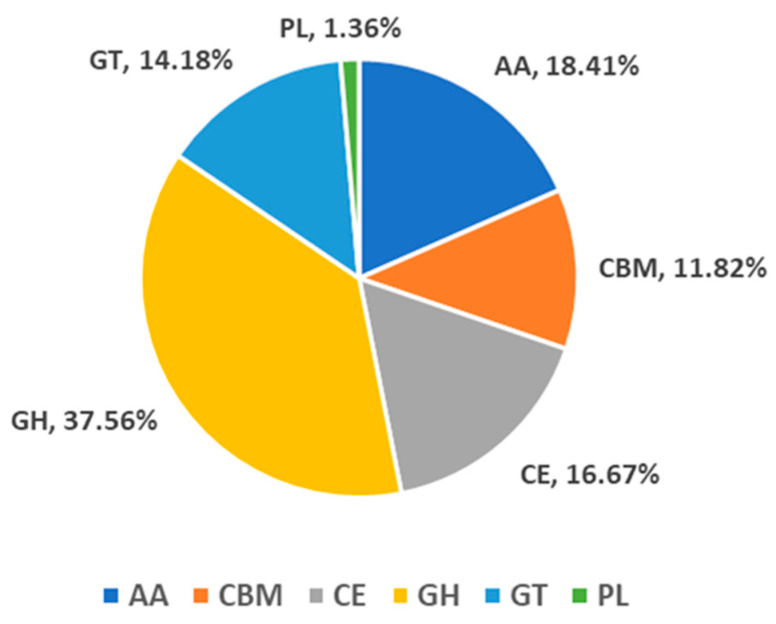
Predicted carbohydrate-active enZYmes of *N. musae* ST1. AA, auxiliary activity; GH, glycoside hydrolase; GT, glycosyl transferase; PL, polysaccharide lyase; CE, carbohydrate esterase; CBM, carbohydrate-binding module.

We annotated 722 genes in the CAZy database. Plant pathogenic fungi degrade plant polysaccharides by producing a large number of carbohydrate-active enzymes (CAZymes), which are classified into five categories based on their functions: glycosyl transferases (GTs), glycoside hydrolases (GHs), carbohydrate esterases (CEs), polysaccharide lyases (PLs), and auxiliary activities (AAs). In addition, the database also includes enzymes related to carbohydrate-binding modules (CBMs). In *N. musae* ST1, the predicted CAZymes comprised 302 GHs, 148 AAs, 134 CEs, 114 GTs, 95 CBMs, and 11 PLs (Figure A2).

### 3.5. Pathogenicity System Analysis

#### 3.5.1. Analysis of PHI

**Figure 7:**
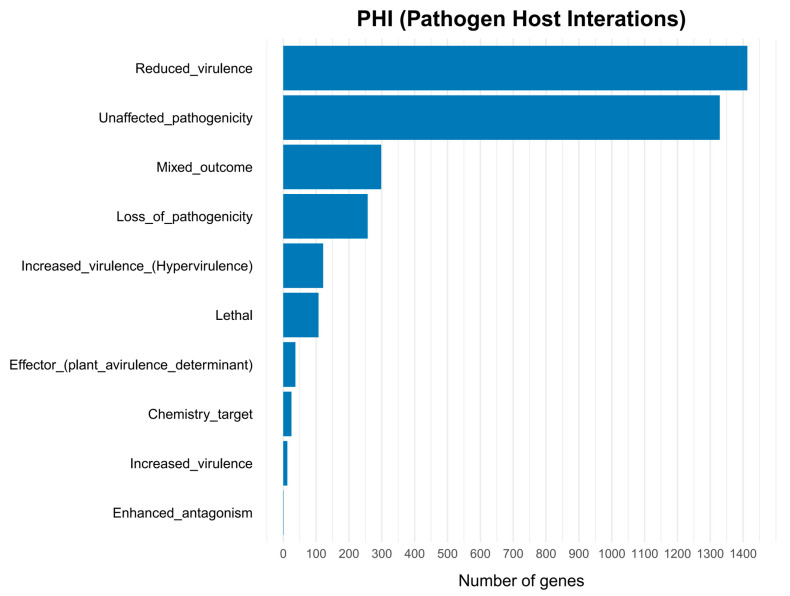
PHI functional classification of proteins encoded in the *N. musae* ST1 genome.

We used the pathogen host interaction (PHI) database to annotate the pathogenicity-related genes in the genome of *Nigrospora*. A total of 3600 genes were annotated in the PHI database (Table 1), accounting for 29.84% of the predicted genes. Among the genes, 121 genes were associated with hypervirulence, 107 genes were related to lethality, 37 genes were associated with effector function, and 12 genes were associated with increased virulence. The largest group consisted of genes linked to reduced virulence (1413 genes), followed by unaffected pathogenicity with 1329 genes, mixed effects with 298 genes, loss of pathogenicity with 257 genes, chemistry target with 25 genes, and enhanced antagonism with 1 gene (Figure 7). *Gibberella zeae* (993 genes) and *Magnaporthe oryzae* (624 genes) had the highest number of homologous genes related to virulence.

#### 3.5.2. Database of Fungal Virulence Factor (DFVF) Annotations

Fungal Virulence Factors are widely involved in fungal pathogenicity processes such as host tissue infection, immune evasion, and toxin production. A total of 2503 genes were annotated in the DFVF database, accounting for 20.75% of the predicted genes.

#### 3.5.3. Transporter Classification Database (TCDB) Annotations

Transporters are proteins on the cell membrane that regulate the influx and efflux of substances, thereby participating in nutrient uptake and energy metabolism. In fungi, certain transporters are closely related to fungal resistance and pathogenicity, as well as fungal growth and spore formation [74,75,76,77]. For example, disruption of the ABC1 gene in *Magnaporthe grisea* reduces its infectivity toward rice and barley [78]. In this study, a total of 125 genes were annotated in the TCDB, representing 1.04% of the predicted genes.

#### 3.5.4. Cytochrome P450 (CYP450) Genes

Cytochrome P450 enzymes constitute a superfamily of structurally and functionally related isoenzymes involved in cell detoxification, xenobiotic degradation, and biosynthesis of secondary metabolites [79]. A total of 585 CYP450 genes were predicted in the *N. musae* ST1 genome (Table 1).

### 3.6. Protein Subcellular Localization Analysis

Effector proteins are a class of specialized proteins secreted by pathogens that can promote the infection and colonization process by targeting the host’s immunity signaling or metabolic pathways [80]. Subcellular localization prediction identified a total of 1462 proteins with signal peptides, 2713 transmembrane proteins, and 1071 secreted proteins. Finally, the secreted proteins were further analyzed with EffectorP, and 197 effector proteins were predicted (Table 1).

**Figure 8:**
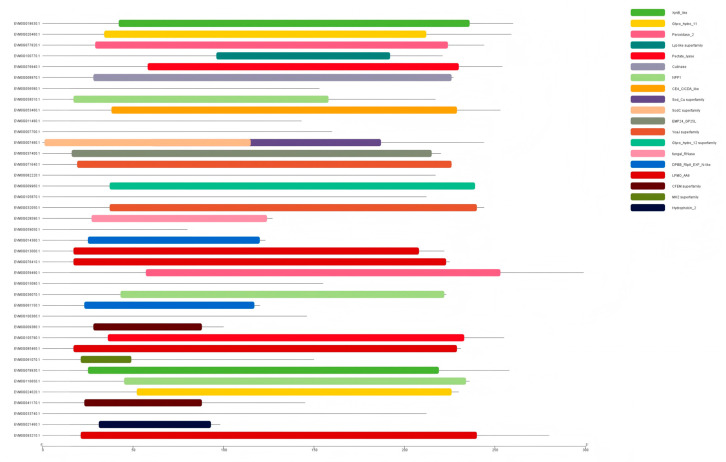
Distribution of candidate effector domains for *N. muse* ST1.

To suppress pattern-triggered immunity (PTI) elicited by plant pathogen-associated molecular patterns (PAMPs), plant pathogens often secrete effectors and enhance pathogenicity by regulating host metabolism and defense response, leading to effector-triggered susceptibility (ETS) [81]. Most known fungal effectors are characterized by signal peptides, low molecular weight, and rich cysteine residues [82]. After filtering the 197 predicted effectors to exclude proteins exceeding 300 amino acids, 39 candidate pathogenicity-related effectors were obtained (Table A3). Among these, 20 were hydroxy proteins, while the remainder included acetylxylan esterase, endoglucanase-like proteins, endo-1,4-beta-xylanase B, and related enzymes. Meanwhile, we analyzed the functional structure of 39 candidate effector proteins, including 30 proteins with domains and 9 proteins lacking domains (Figure 8).

**Figure 9:**
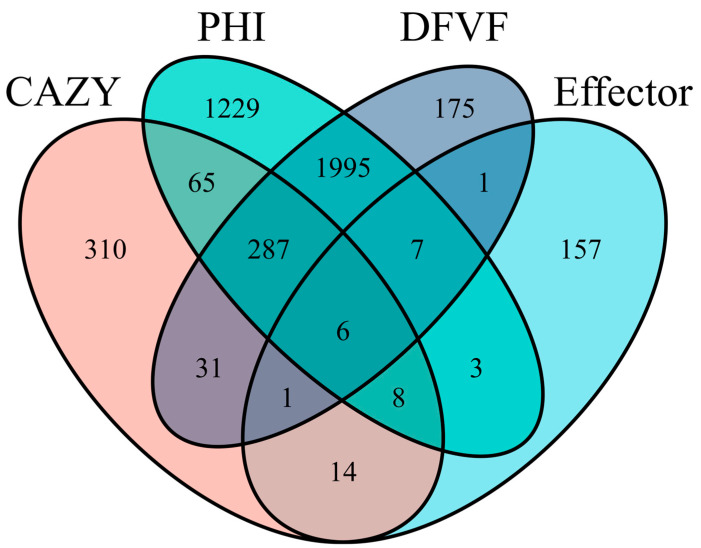
Venn diagram of genes encoding CAZymes, PHI, DFVF and effector.

We conducted an integrated analysis of CAZymes, PHI, DFVF pathogenic gene-related databases, and effector proteins. The Venn diagram (Figure 9) showed that six genes were shared by CAZymes, PHI, DFVF, and the secretome. Among the six secretory CAZyme genes, three genes encoded β-1,4-xylanases, two of which were endo-1,4-β-xylanases involved in xylan degradation, two genes encoded pectinate lyases that catalyze the hydrolysis of polygalacturonic acid, and one gene encoded a cutinase that catalyzes the hydrolysis of cutin.

### 3.7. Comparative Genomic Analysis of Other Nigrospora Species

Among the currently available genome-annotation studies for *Nigrospora*, only three pathogenic species, *N. oryzae* ZQ1, *N. oryzae* GZL1, and *N. sphaerica* ZJJ_C1, were selected. In addition, the genomes of five plant pathogenic fungi of the same genus, *N. guilinensis* LC3481, *N. hainanensis* LC7030, *N. lacticolonia* LC3324, *N. osmanthi* Z1, and *N. pyriformis* LC2045, were selected from NCBI for comparison and analysis with the genome of *N. musae* ST1. Substantial variation was observed among these species in genome size, contig N50, GC content and other genomic features (Table 3).

**Figure 10:**
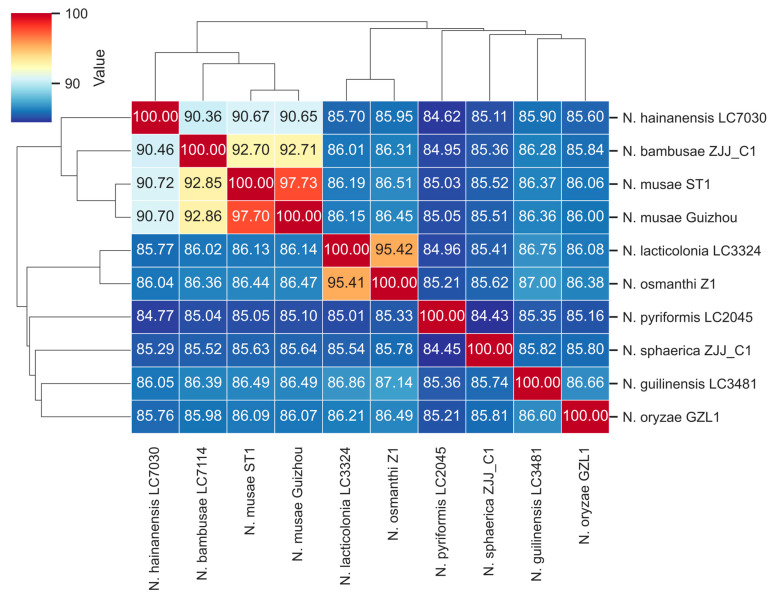
The average nucleotide identity (ANI) values based on the fastANI-algorithm-generated matrix for *Nigrospora* genomes. The clustering was constructed using the Euclidean distance matrix.

Whole-genome alignment and calculation of the average nucleotide identity (ANI) revealed pairwise similarities among *Nigrospora* species ranging from 97.73 to 84.43%. As illustrated in Figure 10, *N. musae* ST1 exhibited low genomic similarity (84.43–86.49%) with *N. lacticolonia* LC3324, *N. osmanthi* Z1, *N. pyriformis* LC2045, *N. sphaerica* ZJJ_C1, *N. guilinensis* LC3481, and *N. oryzae* GZL1. In contrast, *N. musae* ST1 showed high genomic similarity (90.36–97.73%) to *N. hainanensis* LC7030, *N. bambusae* LC7114, and *N. musae* Guizhou. The similarity between *N. musae* ST1 and *N. musae* Guizhou was as high as 97%, indicating that *N. musae* ST1 and *N. musae* Guizhou belong to the same species. Collectively, the results above confirmed that *N. musae* ST1 belongs to the genus *Nigrospora* according to the current classification.

**Figure 11:**
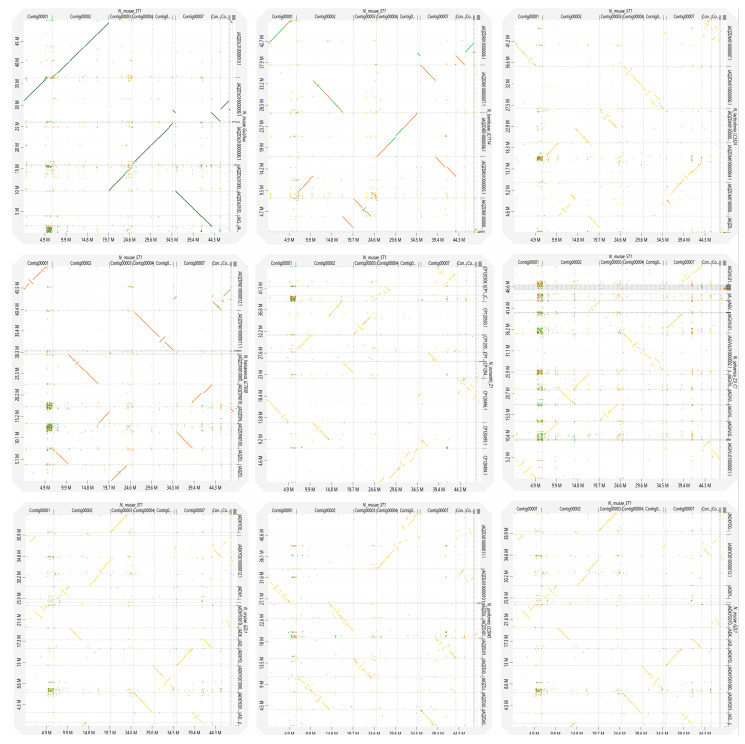
The visualization of the whole-genome LAST alignment of complete and genome-level *Nigrospora* species genome assemblies available in the NCBI Genome database (vertical axis) to obtain a complete *N. musae* ST1 genome assembly (horizontal axis). Orange indicates direct alignments, and blue indicates reverse alignments.

To investigate the genomic synteny and evolutionary relationships within *Nigrospora*, we performed synteny analysis between *N. musae* ST1 and nine closely related species, including *N. bambusae* LC7114, *N. guilinensis* LC3481, *N. hainanensis* LC7030, *N. lacticolonia* LC3324, *N. musae* Guizhou, *N. oryzae* GZL1, and *N. pyriformis* LC2045 (Figure 11). The synteny plots revealed that *N. musae* ST1 shared the longest and most continuous syntenic blocks with *N. musae* Guizhou, indicating strong conservation of genome structure between these two isolates. In contrast, syntenic blocks between *N. musae* ST1 and other species were relatively short and fragmented, suggesting significant genomic structural divergence. These findings provide important genomic-level insights into the evolutionary dynamics and phylogenetic relationships within *Nigrospora*.

## 4. Discussion

Genomic information is fundamental for elucidating the pathogenicity of pathogenic fungi. At present, genome assemblies for 16 *Nigrospora* species are available in NCBI, representing different saprophytes, plant endophytes, plant pathogens, and ecotype-specific strains [85]. Among them, *N.oryzae*, *N.sphaerica* and *N. musae* pose a serious threat to rice, *Akebia trifoliata*, *I. polycarpa*, and other cash crops [12,86]. Despite their agricultural importance, genomic resources for *Nigrospora* remain limited. In this study, the whole genome of the pathogenic fungus *N. musae* ST1 causing *I. polycarpa* leaf spot disease was sequenced and assembled. The assembled genome size of *N. musae* ST1 was 49.26 Mb, and a total of 12,125 coding genes were predicted. There were significant differences in genome size, GC content, gene cluster type, and number compared with other *Nigrospora* species that have been previously characterized. Notably, even when compared with *N. musae* Guizhou, another strain of the same species, *N. musae* ST1, exhibited pronounced differences in the types and number of secondary metabolism gene clusters. Such differences may arise from variations in sequencing technologies or reflect distinct life histories, metabolic potentials and host adaptation strategies due to the different regulatory genes and metabolic pathways in isolates from disparate geographic or ecological origins.

Fungal carbohydrate-active enzymes are closely related to the pathogenicity of pathogens. The plant cell wall is the first barrier for plants to resist pathogens. Pathogens infect plant cells by releasing and secreting a large number of cell wall-degrading enzymes to obtain nutrients [87]. These plant cell wall-degrading enzymes include carbohydrate esterases (CEs), polysaccharide lyases (PLs), and glycoside hydrolases (GHs) [88]. In this study, the number of *N. musae* ST1 glycoside hydrogenases (GHs) was the largest (302), accounting for 41.8% of the total number of CAZyme genes, significantly higher than the number of polysaccharide lyases (PLs) (11) and carbohydrate esterases (CEs) (134). *N. musae* ST1 possesses a high number of carbohydrate-active enzymes, indicating its strong capacity for complex carbohydrate degradation and glycosylation. Among them, the abundance of glycoside hydrolases was significantly higher, which is similar to oomycetes such as *Phytophthora* and *Phytopythium*. The abundance of carbohydrate-active enzymes contributes to efficient and rapid degradation of carbohydrates, and also enhances pathogenicity and host adaptation [89,90,91]. Additionally, *N. musae* ST1 exhibited a notably higher number of genes in the GH61 family compared to other glycoside hydrolase families. The proteins encoded by these genes can significantly enhance lignocellulose hydrolysis [92]. Lignocellulose is a complex polymer composed of cellulose, hemicellulose, and lignin, which provides structural reinforcement to the plant cell wall. The GH61 gene family can efficiently degrade lignocellulose in the plant cell wall and promote plant infection by *N. musae* ST1. For example, members of the GH61 family can serve as key pathogenicity factors. In *Pyrenophora graminea*, deletion of the *PgGH61-2* gene resulted in severely reduced pathogenicity and impaired carbon source utilization [93]. Due to their high catalytic efficiency in depolymerizing recalcitrant substrates, GH61 enzymes can be used for cellulose conversion in biofuel production [94]. *N. musae* ST1 may overcome physical barriers by efficiently degrading plant cell walls rich in lignocellulose. Notably, the number of polysaccharide lyases identified in the *N. musae* ST1 strain was extremely low, and PLs primarily target pectin. The pectin degradation function of *N. musae* ST1 during plant infection may be mediated by members of certain glycoside hydrolase gene families. For instance, GH28 family enzymes encode polygalacturonases, rhamnogalacturonases, and xylogalacturonases that can degrade pectin in plants [95].

We annotated and analyzed genes related to pathogenicity. Genome-wide annotation identified 3600 genes associated with pathogen–host interactions, divided into 10 different categories (Figure 7). This reflects the redundant genetic buffering mechanisms in fungal genomes and the complexity of virulence regulation [96]. A total of 2503 genes were annotated as Fungal Virulence Factors, which play widespread roles in host tissue invasion, immune evasion, and toxin production [62]. A total of 585 CYP450 genes were annotated; these enzymes participate in cellular detoxification, degradation of exogenous substances, and synthesis of secondary metabolites [79]. These genes may regulate toxin synthesis and inhibit host defense responses to enhance pathogenicity. Protein subcellular localization analysis predicted 1462 signal peptides, 2713 transmembrane proteins, and 1071 secreted proteins, from which 197 effector proteins were predicted. Integrated analysis across CAZymes, PHI, DFVF, and the secretome revealed six secreted carbohydrate-active enzymes involved in the degradation of plant structural components and the infection of plants, including cutinases, 1,4-β-xylanases, pectate lyases, and glycoside hydrolase 61. Cutinases can catalyze the hydrolysis of cutin in the plant cuticle, helping pathogenic bacteria break the physical barrier and penetrate the plant epidermis. For example, downregulation of cutinase genes following loss of the transcription factor *Fv*CTF1α in Fusarium verticillioides reduced pathogenicity [97]; Xylan, the main component of plant hemicellulose and cell wall structure, is cleaved at β-1,4-glycosidic bonds by 1,4-β–xylanases; loss of GH11 family genes in *Neostagonosporella sichuanensis* diminished virulence [98]. Pectinate lyases cleave the α-1,4-glycosidic bond in polygalactonic acid through β elimination reaction, disrupting pectin integrity in the cell wall. The pectate lyase gene of *Streptomyces galilaeus* is significantly upregulated during pathogenesis, and plant susceptibility is markedly reduced when this gene is disrupted [99]. The pathogenicity of *Fusarium graminearum* decreased after deletion of different pectate lyase genes [100]. However, in *Peronophythora litchii*, pectate lyase has dual effects of inducing immunity and inhibiting pathogenesis [101], indicating that the functions of pectate lyases in different pathogenic bacteria are different. This functional divergence may arise from the complex regulatory networks shaped during host–pathogen co-evolution. For instance, while pectate lyases enhance pathogen invasion, they can also be targeted by the host immune recognition system. Although the PlPeL1 protein of *Peronophythora litchii* is essential for full virulence, it can be bound by the immune regulatory factor *Lc*PIP1 secreted by litchi, thereby attenuating the induction of host susceptibility [101]. Overall, these enzymes likely represent key contributors to *N. musae* ST1 infection and disease causation, providing valuable candidate genes for future functional validation of pathogenicity factors.

We further analyzed the predicted effector proteins. A total of 39 candidate effectors were identified, comprising 25 apoplastic and 14 non-apoplastic effectors. It is worth noting that in the apoplastic effectors, we identified enzymes related to cell wall degradation, such as glycoside hydrolases, acetylxylan esterases, endoglucanase-like proteins, Endo-1, 4-beta-xylanase B, and putative glycoside hydrolase family 61 proteins. These enzymes are directly involved in the degradation process of plant cell walls. They degrade the physical defense barrier of plant cells by degrading cellulose, hemicellulose, and xylan, facilitating pathogen entry, colonization, and nutrient acquisition [97,98,102]. In addition, a hypersensitive response-inducing protein was identified, capable of triggering host cell death and enhancing plant disease resistance [103]. Although these effector-related genes have been studied in other pathogens, their functions can vary substantially across species. For example, the glucosidase GH12 gene, which is one of the important virulence factors, can induce strong necrosis and resistance in *Phytophthora* [102]. However, the glucosidase protein in *Cytospora chrysosperma* of *PopulusL* can induce plant immunity, but has no effect on pathogenicity [104]. At present, there are few studies on the pathogenicity mechanisms of *Nigrospora* plant pathogenic fungi, and it is essential to further clarify the roles of *N. musae* ST1 effectors in virulence, host specificity, or environmental adaptation.

Compared with other *Nigrospora* species, *N. musae* ST1 possesses the highest number of secondary metabolite biosynthetic gene clusters, followed by *N. musae* Guizhou and *N. sphaerica* ZJJ_C1. Moreover, the genomes of fungi within this genus are all rich in T1PKS and terpene gene clusters (Figure A1). T1PKS gene clusters are responsible for catalyzing the biosynthesis of polyketide compounds, while terpene gene clusters produce terpenoids. Both types of compounds play critical roles in fungal ecological interactions, environmental adaptation, and pathogenicity [105]. For instance, various T1PKS gene clusters identified in the genome of *Xanthoria elegans* contribute to the synthesis of UV-absorbing compounds that support survival in extreme environments [106]. In the plant pathogenic fungus *Cytospora chrysosperma*, knockout of the terpene synthase gene *CcPtc1* significantly reduced fungal virulence and altered terpenoid metabolite profiles, highlighting the importance of terpene biosynthesis in pathogenicity [107]. Notably, *N. musae* ST1 and the conspecific strain *N. musae* Guizhou exhibit significant differences in the types and numbers of gene clusters (Figure A1). Such intraspecific variation in gene clusters is a typical characteristic of fungal genomic plasticity. The expansion and contraction of secondary metabolite gene clusters are major drivers of fungal evolution and can occur through gene duplication, transposon-mediated duplication, and horizontal gene transfer. For example, in the birch pathogen *Inonotus obliquus*, lineage-specific whole-genome duplication events and subsequent retention of cytochrome P450 genes and small-scale duplications within biosynthetic clusters have greatly diversified terpenoid biosynthesis [108]. Similarly, in the plant pathogen *Fusarium*, evolutionary analysis of the trichothecene biosynthesis gene cluster further elucidated the molecular mechanism by which new genes are recruited into existing clusters through gene rearrangements, leading to gene cluster expansion [109]. Therefore, the pronounced differences in gene clusters between *N. musae* ST1 and *N. musae* Guizhou may reflect genomic differentiation driven by distinct ecological origins, host associations, or microenvironmental pressures. Through such dynamic genomic remodeling, these fungi may have evolved unique chemical repertoires and pathogenic strategies.

There are many types of fungal secondary metabolites, most of which belong to polyketones [110]. These compounds induce cell death by destroying the host cell membrane structure, interfering with mitochondrial function, and inhibiting oxidase activity, thereby enhancing pathogenicity [110,111,112,113,114]. In this study, a total of 77 secondary metabolite biosynthetic gene clusters (bgcs) were predicted in the genome of *N. musae* ST1. Among them, the number of T1PKS gene clusters was the largest (29). Given their central role in polyketide biosynthesis, these clusters may contribute to the strong pathogenic potential of *N. musae* ST1. For example, the PKS gene cluster of *Cercospora sojina* is involved in the early infection process [115], and *Alternaria spp* fungi can produce a variety of host-specific polyketide toxins [116]. In addition to being involved in fungal pathogenicity, polyketide secondary metabolites can also support fungal survival under adverse conditions, such as DHN-melanin, which protects against environmental stress [117,118]. In addition, the non-ribosomal peptide synthase (NRPS) gene cluster may promote the synthesis of non-ribosomal peptide compounds and affect the pathogenicity and spore formation of pathogens [119,120]. Three gene clusters exhibited 100% similarity with known secondary metabolite gene clusters responsible for producing alternapyrone, dimethylcoprogen, and serinocyclin A/serinocyclin B. Among them, alternapyrone—whose biosynthesis is regulated by a T1PKS gene cluster—exhibits phytotoxicity and inhibits wheat seed germination by targeting early embryonic development [75]. Furthermore, it also demonstrates cytotoxic activity against tumor cells [68], suggesting potential applications in natural product discovery and biomedicine. Notably, 96.10% of BGCs showed no similarity to known gene clusters, indicating that *N. musae* ST1 may produce a wide array of unknown secondary metabolites. The biological activities of these compounds remain unknown and warrant further investigation. Furthermore, among other species in the same genus, *N. musae* ST1 possessed the highest number of secondary metabolite clusters, underscoring its extensive metabolic potential.

Based on whole genome sequences, we compared and analyzed the fungi of the genus *Nigrospora*. The results of ANI analysis showed that the similarity between *N. musae* ST1 and the strain of the same species, *N. musae* Guizhou, was as high as 97%, and the similarity with *N. hainanensis* LC7030 and *N. bambusae* LC7114 was 90.36–92.71%. In contrast, similarity with *N. lacticolonia* LC3324, *N. osmanthi* Z1, *N. pyriformis* LC2045, *N. sphaerica* ZJJ_C1, *N. guilinensis* LC3481, and *N. oryzae* GZL1 was only 84.43–86.49%, which further confirmed the classification status of *N. musae* ST1 as a distinct species within the genus. Collinearity analysis showed that there were extensive genome rearrangements among different species of *Nigrospora*. Even between the strains *N. musae* ST1 and *N. musae* Guizhou, genome structures were not completely conserved, and there were segment inversions and translocation variations. Research on fungal species diversity and host adaptation indicates that fungi often evolve from broad-spectrum to specialized hosts, with genomic plasticity serving as a key mechanism driving this adaptive process [121]. Furthermore, in closely related fungal groups, variations in genomic synteny have been confirmed to be closely associated with host adaptation and the differentiation of pathogenic strategies. Comparative genomic studies of *Colletotrichum* fungi have revealed significant syntenic rearrangements at the genome assembly level among strains of the same species, correlated with the distribution of secondary metabolite gene clusters and differences in gene expression. This indicates that genomic structural variation is an important evolutionary driver for fungi to adapt to different ecological niches [122]. Such structural variation likely represents an important evolutionary strategy for *Nigrospora* fungi to adapt to different hosts and ecological environments. 

## 5. Conclusions

In this study, we performed de novo whole-genome sequencing of *N. musae* ST1 using the PacBio Sequel II platform. In order to gain a better understanding of *N. musae* ST1 genome structure and function, we further predicted CAZymes and annotated PHI, DFVF, TCDB, and P450-encoding genes. These results provide a basis for the study of the pathogenicity genes of the fungus. In addition, based on the large number of secondary metabolite biosynthetic gene clusters identified in this study, we can further explore the role of secondary metabolite gene clusters on the pathogenicity of *Nigrospora*.

## Figures and Tables

**Table 1:** The genomic characteristics of *N. musae* ST1.

	Content	Values
Reads features	SeqNum	348,147
	SumBase (G)	2.731363334
	LenN50	9400
	MeanLen	7845
	MaxLen	42,256
Assembly results	Assembly Size (Mb)	49.25
	Contig Length (bp)	49,259,803
	Contig Number	42
	Contig N50 (bp)	6,617,117
	Contig N90 (bp)	2,194,971
	GC content (%)	56.23
	Gap Number	0
Proprietary database notes	Carbohydrate-Active enZYmes Database	722
	Transporter Classification (TC) System	125
	PHI	3600
	CYtochrome P450 Engineering Database	585
	DFVF	2503
Protein subcellular localization analysis	Signal Peptide	1462
	Transmembrane Protein	2713
	Secreted Protein	1071
	Effector_protein	179

**Table 2:** The assembly evaluation of *N. musae* ST1.

Evaluation Method	Features	Values
evaluation of next-generation sequence data recovery	Library	350 bp
	Mapped (%)	98.93
	Properly mapped (%)	94.56
	Coverage (%)	99.97
	Depth (X)	69.81
Busco assessment	Complete BUSCOs (C)	287 (98.97%)
	Complete and single-copy BUSCOs (S)	287 (98.97%)
	Complete and duplicated BUSCOs (D)	0 (0.00%)
	Fragmented BUSCOs (F)	1 (0.34%)
	Missing BUSCOs (M)	2 (0.69%)
	Total Lineage BUSCOs	290

**Table 3:** Comparison of genomic features among different *Nigrospora* species.

Species	Assembly Level	Genome Size, Mb	BUSCO Completeness	Contig N50, Mb	Predicted Genes	Average Gene Length (bp)	GC Content (%)	Host	Assembly Accession	Sequencing Technology
*N. musae* ST1	Complete	49.25	98.97%	7	12,125	2148.5	56.23	Idesia polycarpa	-	Pacbio Sequel II
*N. musae* Guizhou	Complete	50	-	9.9	-	-	56.5	Plant tissue	GCA_048773425.1	Oxford Nanopore
*N. Oryzae* ZQ1 [83]	Complete	42.09	98.97%	-	10,688	2356	58.83	Rice Leaf	-	Oxford Nanopore
*N. oryzae* GZL1 [84]	Complete	43.41	99.30%	4	10,039	-	58.19	Maize	GCA_016758845.1	PacBio
*N. Sphaerica* ZJJ_C1 [12]	Complete	51.75	99.30%	4.6	11,518	-	56.09	Akebia trifoliata	GCA_018287875.1	PacBio Sequel
*N. Guilinensis* LC3481	Complete	47.5	-	7.6	-	-	57	Plant tissue	GCA_048773485.1	Oxford Nanopore
*N. Hainanensis* LC7030	Complete	50.5	-	7.1	-	-	56	Plant tissue	GCA_048773445.1	Oxford Nanopore
*N. Lacticolonia* LC3324	Complete	45.8	-	8.5	-	-	57.5	Plant tissue	GCA_048773465.1	Oxford Nanopore
*N. osmanthi* Z1	Chromosomes	45.9	-	7	-	-	58	Water Lettuce	GCA_029489945.1	PacBio
*N. pyriformis* LC2045	Complete	45.1	-	4.2	-	-	58	Plant tissue	GCA_048773265.1	Oxford Nanopore
*N. bambusae* LC7114	Complete	47.4	-	9.8	-	-	56	Plant tissue	GCA_048773625.1	Oxford Nanopore

## Data Availability

The read data of Pacbio Sequel II sequencing have been submitted to the NCBI Sequence Read Archive with accession number PRJNA1367030. This Whole Genome Shotgun project has been deposited at GenBank under accession number JBSLLA000000000. The version described in this paper is version JBSLLA000000000.

## Appendix A

**Table A1:** Basic information statistics of the predicted genes.

	Genome Features	Values
Repeat Sequence Prediction	ClassI	1774
	ClassI/DIRS	167
	ClassI/LINE	231
	ClassI/LTR	1
	ClassI/LTR/Copia	222
	ClassI/LTR/Gypsy	1078
	ClassI/PLE|LARD	67
	ClassI/TRIM	6
	ClassI/Unknown	2
	ClassII	570
	ClassII/Crypton	2
	ClassII/Helitron	7
	ClassII/TIR	508
	ClassII/Unknown	53
	Total	2344
Protein-Coding Gene	Number of protein-coding genes	12,063
	Total length of protein-coding genes	25,915,843
	Average length of protein-coding genes	2148.37
	Total exon length	23,439,261
	Average length of exons	658.5
	Number of exons	35,595
	Average number of exons per gene	2.95
	Total length of CDS	18,207,240
	Average length of CDS	523.75
	Number of CDS	34,763
	Average number of CDSs per gene	2.88
	Total length of intron	2,476,582
	Average length of intron	105.24
	Number of introns	23,532
	Average number of introns per gene	1.95
Non-Coding RNA	rRNA	78
	tRNA	332
	other ncRNA	75
Pseudogene	Pseudogene number	17
	Pseudogene size (bp)	65,191
	Average Pseudogene Length (bp)	3834.76
genecluster	The number of gene clusters	77
	Gene cluster total length (bp)	3,225,987
	Gene cluster mean length (bp)	41,895

**Table A2:** Putative biosynthetic gene clusters of *N. musae* ST1 showing similarity to a minimum number of known gene clusters in the biosynthetic gene cluster database.

Region	Type	Location	Length (bp)	Most Similar Known Cluster	Similarity	Similarity (%)
Region 1.2	NRPS-like, T1PKS	2,096,713–2,138,524	41,811	swainsonine	Polyketide	42%
Region 1.5	NRPS-like, indole	4,855,436–4,898,375	42,939	ochrindole A	Other	11%
Region 2.5	NRPS, T1PKS	3,393,293–3,480,198	86,905	equisetin	NRP + Polyketide	45%
Region 2.6	NRPS, T1PKS, indole	3,688,110–3,891,603	203,493	eupenifeldin	Terpene	45%
Region 2.7	T1PKS	4,231,972–4,271,036	39,064	alternapyrone	Polyketide	100%
Region 2.8	indole	4,663,450–4,684,613	21,163	clapurines	NRP	36%
Region 2.15	NRPS, T1PKS	7,819,874–7,872,572	52,698	ilicicolin H	NRP + Polyketide	33%
Region 3.5	T1PKS	3,894,886–3,929,833	34,947	azanigerone A	Polyketide	33%
Region 3.9	T1PKS	4,668,157–4,714,737	46,580	sterigmatocystin	Polyketide	38%
Region 4.1	T1PKS	164,759–210,849	46,090	betaenone A/betaenone B/betaenone C	Polyketide	62%
Region 4.3	T1PKS, NRPS	573,779–624,625	50,846	curvupallide-B	NRP + Polyketide	27%
Region 4.7	NRPS, T1PKS	4,299,088–4,351,954	52,866	burnettramic acid A	Alkaloid + Polyketide:Iterative type I	33%
Region 5.2	NRPS, NRPS-like	1,681,366–1,764,759	83,393	dimethylcoprogen	NRP	100%
Region 6.1	T1PKS	32,900–80,437	47,537	depudecin	Polyketide:Iterative type I	66%
Region 7.13	terpene	4,274,390–4,286,105	11,715	squalestatin S1	Terpene	40%
Region 8.3	NRPS, T1PKS	1,789,072–1,894,582	105,510	serinocyclin A/serinocyclin B	NRP	100%
Region 8.4	T1PKS	2,015,408–2,060,866	45,458	terreic acid	Polyketide	33%
Region 9.1	T1PKS	647,922–690,670	42,748	asperthecin	Terpene	28%
Region 9.2	T1PKS	1,027,400–1,074,012	46,612	naphthalene	Polyketide	33%

**Table A3:** *N. muse* ST1 candidate effectors.

Gene ID	Protein Length	Predicted Effector Proteins	Effector P Prediction	Effector Probability
EVM0G007480.1	244	hypothetical protein	Apoplastic	0.702
EVM0G007700.1	160	hypothetical protein	Non-apoplastic	0.72
EVM0G008970.1	227	acetylxylan esterase	Apoplastic	0.683
EVM0G009380.1	100	CFEM domain-containing protein	Non-apoplastic	0.909
EVM0G009960.1	239	endoglucanase-like protein	Apoplastic	0.828
EVM0G011480.1	143	hypothetical protein	Non-apoplastic	0.582
EVM0G013000.1	220	hypothetical protein	Non-apoplastic	0.69
EVM0G014360.1	123	putative barwin-like endoglucanase protein	Apoplastic	0.575
EVM0G015080.1	155	hypothetical protein	Apoplastic	0.804
EVM0G018630.1	260	hypothetical protein	Non-apoplastic	0.689
EVM0G020460.1	259	hypothetical protein	Apoplastic	0.619
EVM0G021460.1	98	hypothetical protein	Apoplastic	0.59
EVM0G024020.1	230	endo-1,4-beta-xylanase B	Apoplastic	0.803
EVM0G028590.1	127	guanyl-specific ribonuclease F1	Apoplastic	0.675
EVM0G032050.1	244	barwin-like endoglucanase	Apoplastic	0.767
EVM0G033740.1	212	hypothetical protein	Non-apoplastic	0.848
EVM0G036070.1	223	elicitor	Non-apoplastic	0.681
EVM0G037400.1	220	hypothetical protein	Non-apoplastic	0.626
EVM0G041170.1	145	putative cfem domain-containing protein	Apoplastic	0.602
EVM0G053460.1	253	hypothetical protein	Apoplastic	0.648
EVM0G056580.1	153	hypothetical protein	Non-apoplastic	0.581
EVM0G058510.1	217	uncharacterized protein FFUJ_12628	Apoplastic	0.941
EVM0G059050.1	80	hypothetical protein	Apoplastic	0.775
EVM0G059490.1	299	hypothetical protein	Non-apoplastic	0.581
EVM0G061070.1	150	hypothetical protein	Apoplastic	0.897
EVM0G061150.1	120	putative barwin-like endoglucanase protein	Apoplastic	0.552
EVM0G071640.1	226	rare lipoprotein A	Apoplastic	0.624
EVM0G076410.1	225	putative glycoside hydrolase family 61 protein	Apoplastic	0.787
EVM0G076940.1	254	hypothetical protein	Apoplastic	0.681
EVM0G077820.1	244	sterigmatocystin biosynthesis peroxidase stcc	Non-apoplastic	0.613
EVM0G078930.1	258	hypothetical protein	Apoplastic	0.619
EVM0G082220.1	217	uncharacterized protein	Non-apoplastic	0.81
EVM0G085460.1	231	hypothetical protein	Non-apoplastic	0.606
EVM0G093210.1	280	murein transglycosylase	Non-apoplastic	0.709
EVM0G100360.1	146	hypersensitive response-inducing protein	Apoplastic	0.59
EVM0G100770.1	221	putative exo-beta-glucanase protein	Apoplastic	0.709
EVM0G105790.1	255	hypothetical protein	Apoplastic	0.812
EVM0G105870.1	212	hypothetical protein	Apoplastic	0.658
EVM0G116650.1	236	elicitor	Apoplastic	0.559
